# Adherence to artemisinin-based combination therapy for the treatment of malaria: a systematic review of the evidence

**DOI:** 10.1186/1475-2875-13-7

**Published:** 2014-01-06

**Authors:** Kristin Banek, Mirza Lalani, Sarah G Staedke, Daniel Chandramohan

**Affiliations:** 1 Department of Clinical Research, London School of Hygiene and Tropical Medicine, Keppel Street, WC1E 7HT, London, UK; 2 Department of Disease Control, London School of Hygiene and Tropical Medicine, Keppel Street, WC1E 7HT, London, UK

**Keywords:** Malaria, Artemisinin-based combination therapy, ACT, Adherence, Compliance

## Abstract

**Background:**

Increasing access to and targeting of artemisinin-based combination therapy (ACT) is a key component of malaria control programmes. To maximize efficacy of ACT and ensure adequate treatment outcomes, patient and caregiver adherence to treatment guidelines is essential. This review summarizes the current evidence base on ACT adherence, including definitions, measurement methods, and associated factors.

**Methods:**

A systematic search of the published literature was undertaken in November 2012 and updated in April 2013. Bibliographies of manuscripts were also searched and additional references identified. Studies were included if they involved at least one form of ACT and reported an adherence measurement.

**Results:**

The search yielded 1,412 records, 37 of which were found to measure adherence to ACT. Methods to measure adherence focused on self-report, pill counts and bioassays with varying definitions for adherence. Most studies only reported whether medication regimens were completed, but did not assess how the treatment was taken by the patient (i.e. timing, frequency and dose). Adherence data were available for four different ACT formulations: artemether-lumefantrine (AL) (range 39-100%), amodiaquine plus artesunate (AQ + AS) (range 48-94%), artesunate plus sulphadoxine-pyrimethamine (AS + SP) (range 39-75%) and artesunate plus mefloquine (AS + MQ) (range 77-95%). Association between demographic factors, such as age, gender, education and socio-economic status and adherence to ACT regimens was not consistent. Some evidence of positive association between adherence and patient age, caregiver education levels, drug preferences, health worker instructions, patient/caregiver knowledge and drug packaging were also observed.

**Conclusions:**

This review highlights the weak evidence base on ACT adherence. Results suggest that ACT adherence levels varied substantially between study populations, but comparison between studies was challenging due to differences in study design, definitions, and methods used to measure adherence. Standardising methodologies for both self-report and bioassays used for evaluating adherence of different formulations across diverse contexts would improve the evidence base on ACT adherence and effectiveness; namely, specific and measurable definitions for adherence are needed for both methodologies. Additionally, further studies of the individual factors and barriers associated with non-adherence to ACT are needed in order to make informed policy choices and to improve the delivery of effective malaria treatment.

## Background

Despite increased support for malaria control over the past decade, the malaria burden remains high in many endemic countries, particularly in sub-Saharan Africa [1]. Prompt treatment with artemisinin-based combination therapy (ACT) targeted towards those confirmed to have malaria is a key malaria control strategy [2,3].

In 2003, less than twenty countries had adopted ACT as the first-line treatment for uncomplicated malaria [4,5]. With the support of donors, specifically the Global Fund to Fight Aids, Tuberculosis and Malaria (GFATM) and the President’s Malaria Initiative (PMI), the number of countries that have deployed ACT has increased dramatically, allowing for treatment to be more widely available [5]. By 2010, 84 countries had adopted ACT, with 60 countries providing ACT free-of-charge to all ages in the public sector and eight have piloted the provision of subsidized ACT in the private sector through the Affordable Medicines Facility – malaria (AMFm) [2,6,7]. Changing anti-malarial treatment policy to ACT is not enough to ensure proper treatment of malaria. Addressing access and targeting of these efficacious treatments is necessary [8], recognizing that improving access to effective drugs does not guarantee patient acceptability and ultimately adherence to the medications [9].

**Figure 1:**
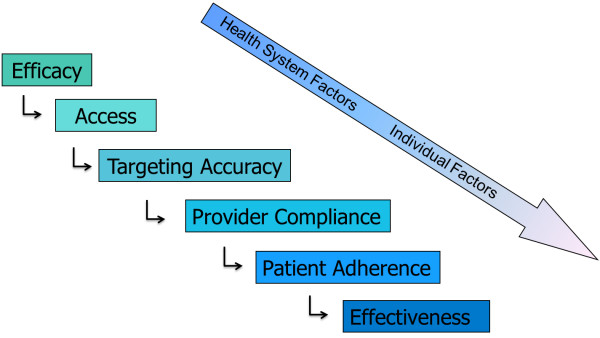
**Treatment effectiveness pathway.** This figure depicts each step along the pathway to malaria treatment effectiveness. At the top of the pathway are the health system factors such as choosing efficacious treatments such as ACT, improving access to those treatments and targeting treatments to those that need it most. The second half of the pathway depicts individual factors that can enhance or disrupt the effectiveness pathway such as provider compliance to treatment guidelines and patient/caregiver adherence to treatment regimens. Source: Original figure courtesy of Marcel Tanner, personal communication 2012 and manuscript published by, The malERA Consultative Group on Health Systems and Operational Research, A Research Agenda for Malaria Eradication: Health Systems and Operational Research. *PLoS Med*, 2011. 8(1): p. E1000397.

The pathway to treatment effectiveness includes a number of factors, each of which contributes to the overall success of an intervention (Figure 1). Each step can independently and collectively impact the overall effectiveness of an anti-malarial treatment regimen. Factors related to poor patient acceptance and adherence not only threaten individual outcomes (recovery), but may lead to higher treatment costs (retreatment) and even resistance [10].

Focusing on the health system challenges to improving access and targeting without addressing factors determining adherence, may ultimately lead to suboptimal health outcomes. Given that resistance to artemisinin compounds has been reported in Southeast Asia [11], and the growing concerns about the spread of resistance and how to contain it, ensuring provider compliance and patient/caregiver adherence to treatment guidelines is even more important.

Strategies that address ‘therapy-related’ factors [12], such as co-packaging anti-malarials into blister packs, have been shown to improve adherence, and reduce the practice of using mono-therapies [9,13,14]. While co-packaging anti-malarial combinations ensures dispensing the correct combination of drugs, it does not reduce the total number of tablets or the frequency the drugs need to be taken. In addition, co-packaging does not necessarily change patient perception or tolerability of individual drugs, and patients may choose to take only one of the medications or not all of the tablets [15-17].

In an effort to overcome the limitations of co-packaged anti-malarial drugs and to improve adherence, several artemisinin-based combinations have been co-formulated; the most common of these are artemether–lumefantrine (AL) and the new co-formulated versions of amodiaquine-artesunate (AQAS) and artesunate-mefloquine (ASMQ).

### Measuring adherence to medications

Despite the large evidence base on adherence to treatment for both chronic and acute disease, no gold standard has been clearly established for measuring patient adherence to medications [18,19]. Adherence can be measured both directly and indirectly. The four most common methods for measuring adherence are: (i) electronic monitoring devices such as the medical event monitoring system (MEMS), (ii) pill counts, (iii) self-report through interviews; and (iv) biological assays [18-22]. Additionally, adherence to medications can be measured by reviewing medical records, patient diaries or by directly observing drug intake (as is often the case for drug efficacy studies).

The default gold standard for measuring medication adherence has been MEMS [18,23]. MEMS containers collect data on the frequency and timing of when the medication container was opened. Traditionally pill counts in the context of chronic disease occurred when patients came back to the health worker and the health worker ‘counted’ the number of remaining tablets thus determining whether the patient was adherent to the treatment protocol [22]. Enumerating the quantity of remaining tablets is rarely used alone, but usually in combination with patient interviews or self-reports. Although MEMS has been adopted as the gold standard for adherence to medications administered for chronic disease, it is not optimal for monitoring adherence to anti-malarial medications.

Bio-assays look at the levels of drug or their metabolites in a biological sample (usually blood or urine) taken from the patient shortly after they have taken their medications. Although this can provide a direct method of measuring whether a medication was ingested, such assays can be costly and are dependent on the availability of laboratory testing and thus are impractical in resource limited settings.

Thus, although a variety of methods have been applied to measure medication adherence, each method has both advantages and disadvantages. Therefore, no clear gold standard exists for measuring adherence for treatment of acute diseases (like malaria).

### Adherence to anti-malarial drugs

In 2005, Yeung and White produced a comprehensive review of 24 studies on how anti-malarials were used by patients [10]. As this review was undertaken in the infancy of the ACT era, two studies looked at artemisinin monotherapy treatments [24,25] and six studies looked at ACT; four in Asia [26-29] and two in Africa [30,31]. Only one study carried out in Uganda looked at a co-formulated ACT (AL) [31]. Results for adherence varied for ACT, ranging from 78% for a three-day regimen of AS + SP in Zambia [30] to maximum of 93% for AL in Uganda [31]. Adherence was found to be generally better when “interventions focusing on provider knowledge and behaviour, packaging and provision of correct dosage” were implemented [10].

Since 2005, ACT, and in particular co-formulated versions of ACT have been scaled up across Africa. However, despite the key role adherence plays in treatment effectiveness, the evidence on adherence to ACT in operational settings is limited. In order to summarize the current evidence base on ACT adherence, a systematic review of current peer-reviewed literature was undertaken. In addition, the methods to measure adherence, definitions of adherence, and factors affecting adherence to ACT were also examined.

## Methods

### Search strategy

A systematic search of the published literature was undertaken in November 2012 and updated in April 2013. Three databases (Medline, Embase and Global Health) were searched using predefined search terms (see Additional file 1: Literature Review Search Strategy). References were imported into the electronic reference manager Endnote and duplicates removed. Bibliographies of manuscripts were searched and additional relevant references identified and, where appropriate, included in the review.

Titles and abstracts were screened for relevance based on the inclusion/exclusion criteria. Studies that reported adherence to malaria treatment were retained for further review. The full texts of the remaining studies were read by two different reviewers to ensure they met the inclusion criteria and to improve the quality of the data extracted. Studies were included if they involved at least one ACT, had primary or secondary data on adherence, were found in a peer reviewed journal, written in English, and published after 1990 and up to April 2013. We included any study that reported measuring adherence and/or levels of adherence to ACT, including effectiveness trials that had measured adherence as a secondary outcome.

### Data extraction and presentation

An electronic matrix was developed in Microsoft Excel prior to the full text review with predetermined characteristics. Studies were evaluated using quality measures adapted from the Critical Appraisal Skills Programme (CASP) [32], STROBE [33,34] and the CONSORT guidelines [35] to facilitate a comparison of quality across studies (see Additional file 2: Quality Assessment of Studies). Studies were independently assessed and information extracted by two reviewers and the findings were compared and compiled. A third reviewer settled any discordance between the initial two reviewers. Due to a lack of homogeneity among the studies a meta-analysis of the adherence data was not possible. This review provides a description of the study characteristics, methods and their findings presented by drug combination and study design.

## Results

**Figure 2:**
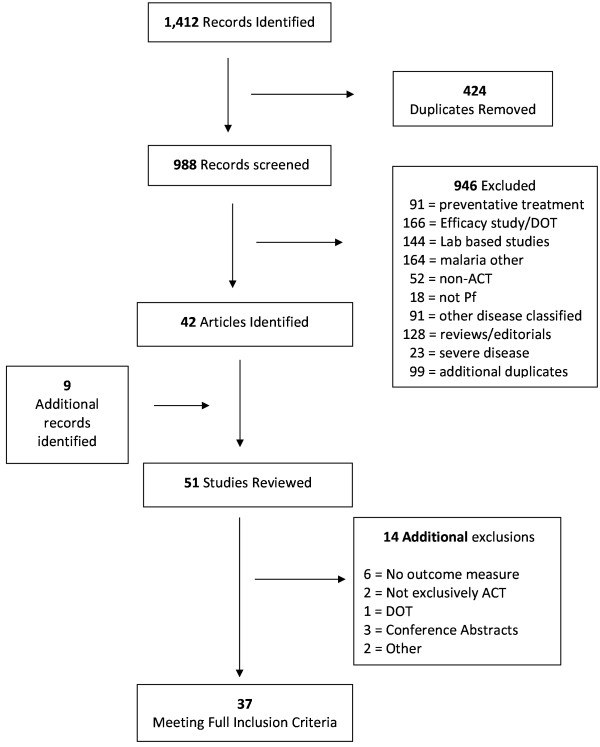
**Systematic review process.** Flow diagram (adapted from PRISMA) describing the systematic review of the literature on ACT adherence.

The search yielded 1,412 records, 424 of which were duplicate records and were subsequently removed (Figure 2). The titles and abstracts of the remaining records (988) were screened and 42 articles were found to be eligible for a full-text review. An additional nine studies were identified from the reference lists and were also included in the review. From the review of the full-text versions of the 51 articles, 14 studies were found to not meet the inclusion criteria. The search yielded 37 articles that evaluated patient adherence to ACT, which were subsequently reviewed and summarized.

### Definitions used for ACT adherence

The definition of patient adherence was not standardized across studies, however the majority used a variation of the same definitions first used by Depoortere and Fogg [30,31], and defined adherent patients/caregivers as those who reported to have taken the treatment as recommended (in terms of timing and dosage) with no tablets remaining. In the instance where the packaging was not available the patients were classified as probably adherent.

Twenty-nine studies reported whether patients/caregivers took/administered all of the prescribed medication (using pill count, self-report or both methods), but did not report exactly how the medication was taken (i.e. timing, frequency and dose). Twelve of those studies expanded this definition of adherence and also investigated both the duration and timing of each dose in order to determine whether the drug was taken as recommended, but these were limited to studies investigating AL [36-47]. Some therapeutic effectiveness studies measured drug metabolites to determine if a treatment had been taken, thus the definition of adherence was only based on the presence or levels of drug metabolites in the blood [27,48].

### Methods of measuring ACT adherence

All four methods commonly used to measure adherence to medications were used to measure ACT adherence. Self-report from patients/caregivers alone [39-41,46,49-54] or in combination with pill counts was found to be the primary method of assessing adherence to ACT treatment [30,31,36-38,42-45,47,48,55-65]. Only one study in Ghana reported using self-report alone [66].

A single study in Malawi reported using MEMS to measure ACT adherence [67]. The use of MEMS was limited to a subset of patients and was subject to availability of the bottles. This measurement was used in combination with patient questionnaires and biological assays. Patients self-reported 100% adherence to AL, but with the MEMS only 92% were found to be adherent, suggesting that self-report might overestimate adherence.

Eight studies reported using biological assay methods, including five that evaluated lumefantrine blood concentrations [31,45,46,67,68] and three in which bio-assays were used for studies involving AS + MQ and comparator drugs [26,27,50]. Five of the studies used bioassay in combination with self-report [31,46,50,67,68].

### Adherence to ACT

#### Artemether-lumefantrine

Almost half of the studies (17) looked at patient adherence to AL (Table 1), fourteen of which were conducted in East or Southern Africa [30,31,36-38,43,44,46,49,51],[57,63,64,68]. Only one study was conducted in West Africa (Ghana) within a study looking at the feasibility of Home Management of Malaria [42]. Two studies were conducted in Asia, one in Myanmar [47] and the other in Bangladesh [45].

**Table 1:** Studies that measure adherence to AL

**Study design**	**Study author**	**Country**	**Study year**	**Population**	**Sample size**	**Measurement method**	**Adherence**
**Cross**-**sectional**	Barnes [49]	South Africa	2002	All ages	239	Self-report	96.0%
	Simba [46]	Tanzania	2008	3-59mo	467	Bioassay (blood levels) plus self-report	88.3%
**Prospective observational**	Depoortere [58]	South Sudan	2002	6-59mo	107	Pill counts & self -report (questionnaire)	59.1%
	Fogg [31]	Uganda	2002	<5 yrs 5-14 yrs 15 + yrs	210	Pill counts & self-report (questionnaire) & bioassay	90.0%
	Ngasala [68]	Tanzania	2007	3-59mo	177	Bioassay (D7 lumefantrine levels)	37%
	Kabanywanyi [36]	Tanzania	2008	<13 yrs 13 + yrs	552	Pill counts & self -report (questionnaire)	89.2%
	Lemma [43]	Ethiopia	2008	>2mo	180	Pill counts & self -report (questionnaire)	38.7%
	Mace [38]	Malawi	2009	6-59mo 5-17 yrs 18 + yrs	868	Pill counts & self -report (questionnaire)	65.0%
	Ogolla [44]	Kenya	2009	12-59mo	73	Pill counts & self -report (questionnaire)	75.8%
	Lawford [37]	Kenya	2009	<15 yrs 15 + yrs	918	Pill counts & self -report (questionnaire)	64.1%
	Kalyango [63]	Uganda	2011	4-59mo	1256	Pill counts & self -report (questionnaire)	99.2% (I)^iii^
							98.5% (C)
	Zaw Win [47]	Myanmar	2012^iv^	All ages	248	Pill counts & self -report (questionnaire)	89.5%
**Pre-post intervention study**	Chinbuah [42]	Ghana	2004/ 2005	6-59mo	363	Pill counts & self -report (questionnaire)	92.5%^v^
	Kangwana [51]	Kenya	2008/ 2009	3-59mo	3,288b; 3,182a	Self-report	53.1% Before 67.0% After^vi^
**RCT**	Mubi [64]	Tanzania	2006	All ages	2156	Pill counts & self -report (questionnaire)	99.3% CD^vii^
							97.4% RDT
	Rahman [45]	Bangladesh	2006/ 2007	>2 yrs	320	Pill counts & self -report (questionnaire) & bioassay	93.1%^viii^
	Cohen [57]	Uganda	2009	All ages	395	Pill count or Self-report	65.8%

^i^Self-report only. The lumefantrine levels were not found to be significantly different between those that adhered vs. those that did not adhere.

^ii^Based on a cut-off of 280 ng/ml. Only 37% had > 280 ng/ml.

^iii^(I) = intervention and (C) = combination.

^iv^Year published.

^v^Although described as a pre-post intervention study, adherence data was only provided for the post-intervention phase.

^vi^Numbers presented are for the Intervention group. The control group was 40.5% before/49.4% after. There was no significant difference found between the two groups during the post survey.

^vii^CD = Clinical Diagnosis Group; RDT = Rapid Diagnostic Test Group.

^viii^Non-Directly Observed Treatment.

Levels of adherence for AL ranged from as low as 38.7% in Ethiopia [43] to 96.0% in South Africa [49]. Of the 17 studies evaluating AL, two used cross-sectional household surveys [49,69], three were randomized controlled trials [45,57,64], two were pre-post intervention designs [42,51] and 10 used a prospective observational design [31,36-38,43,44,47,58,63,68]. The two cross-sectional studies, one conducted in South Africa and another in Tanzania found adherence rates to be 96.0% and 88.3% respectively [49,69]. A RCT in Tanzania and another RCT in Bangladesh, both found adherence levels for AL to be greater than 90% [45,64]. In a third RCT in Uganda, patients were blinded to potential follow-up, and adherence was found to be only 65.8% [57]. Pre-post intervention designs were used in Ghana and Kenya with varying adherence rates post intervention (92.5% vs. 67.0% respectively) [42,51].

Despite similarities in measurement methods and contexts for the ten prospective observational studies, adherence measurements were inconsistent [31,36-38,43,44,47,58,63,68]. Ngasala *et al*. found adherence to be as low 37% in Tanzania using blood lumefantrine levels [68] and Lemma *et al*. in Ethiopia found similar results (38.7%) using self-report and pill counts [43]. On the upper end of the spectrum adherence levels measured as high as 98.5% in Uganda and 89.5% in Myanmar both of which used self-report and pill counts [47,63].

Two studies presented day-7 lumefantrine levels to validate AL intake and correlate with treatment outcomes [67,68]. Bell *et al*. found the median day-7 lumefantrine level to be 214 ng/ml, higher than their reference value (<175 ng/ml) for adequate treatment [67]. However, 4/167 samples were found to have below the lower limit of quantification for the assay and were exclude, but this was not linked by the authors to non-adherence. Ngasala *et al*. also reported median day-7 lumefantrine levels (205 ng/ml), but used a <280 ng/ml as the reference cut-off level and did not correlate lumefantrine levels with adherence [68].

Two additional studies compared lumefantrine blood levels between patients that had adhered to treatment and those that were considered non-adherent. In Uganda, day-3 lumefantrine levels in patients were 3.19 μg/ml in adherent compared 2.76 μg/ml in non-adherent patients, but the difference was not found to be statistically significant (p = 0.46) due to the limited sample size for the non-adherent group [31]. Simba *et al*. also found no significant difference (p-value not reported) in median blood lumefantrine concentrations on day-7 in patients that adhered (286 nmol/l) compared to those that did not adhere (261 nmol/l) [46].

A study in Bangladesh assessed day-7 lumefantrine levels for both validating AL intake and to compare adherence to non-adherence. Lumefantrine concentrations were not found to be different between patients receiving directly observed treatment (DOT) (860 ng/ml) versus patients with non-directly observed treatment (NDOT) (671 ng/ml) (p = 0.56) [45]. Furthermore, blood concentrations were also not significantly lower on day 7 in patients that did not adhere to treatment (680 ng/ml) compared to those that had adhered (626 ng/ml) (p = 0.31), as a result of the small number in the non-adherent group.

#### Amodiaquine plus artesunate

The combination amodiaquine plus artesunate (AQ + AS) was investigated in seven studies (Tables 2 and 3) [39,40,56,60,61,65,66]. Reported adherence varied, with a minimum of 48% in Sierra Leone [61] to a maximum of 93% in Ghana [40]. Only two studies (one in Benin and one in Madagascar) have evaluated co-formulated AQAS and the reported adherence was 91% and 83%, respectively [60,65].

**Table 2:** **Studies that measure adherence to AQ** + **AS**

**Study design**	**Study author**	**Country**	**Study year**	**Population**	**Sample size**	**Measurement method**	**Adherence**
**Cross**-**sectional**	Beer [56]	Zanzibar	2006/2007	<5	210	Pill counts & self–report (questionnaire)	77.0%*
**Prospective observational**	Gerstl [61]	Sierra Leone	2008	All patients ≥ 1 year	118	Pill counts & self-report (questionnaire)	48.3%
	Ratsimbasoa [65]	Madagascar	2008/2009	<5	543	Self-report	90.0%**
**RCT**	Asante [66]	Ghana	2009*	15+	401	Pill counts	95.7% (S)***
							92.6% (U)

*Range 29-100%.

**Amodiaquine-artesunate co-formulated/fixed-dose combination.

***(S) = supervised; (U) = unsupervised.

**Table 3:** Studies that measure adherence as comparative studies

**Study design**	**Study author**	**Country**	**Study year**	**Population**	**Sample size**	**Measurement method**	**Drugs**	**Adherence**
**RCT**	Bell [67]	Malawi	2004-2006	>6 mo	841	Bioassay; self-report	AL	100% SR 92.0% MEMS
						(questionnaire); MEMS*	CPD	99.2% SR 90.6% MEMS
							SP	100% DOT
	Dunyo [59]	Gambia	2004	6mo - 10 yrs	1238	Pill Counts & self-report (questionnaire)	AL	67.0%
							CPD	94.0%
	Faucher [60]	Benin	2007	<5 yrs	240	Recovery of drug blisters	AL	83.0%
							AQAS	91.0%
						(pill-count)		
							SP	100%*
	Achan [55]	Uganda	2007/2008	6-59 mo	175	Pill Counts & care giver	AL QNN	94.5% 85.4%
						self-report (questionnaire)		
**Cross-sectional**	Ajayi [39]	Ghana Uganda Nigeria	2008**	6-59 mo	244	Self-report:	AL	Composite 94%
						(timing, # doses, # of days)	AQ + AS	
	Ajayi [40]	Ghana Uganda Nigeria	2008**	6-59 mo	1096	Self-report:	AL	Composite 85%
						(timing, # doses, # of days)	AQ + AS	
	Alba [41]	Tanzania	2004-2008	All ages	32***	Self-report:	AL	69.0%
						(timing, # doses, # of days)	SP	84.0%
							QNN	0%
							Composite	51.0%

*SR = Self-report; MEMS = Medical Event Monitoring System.

**In Ghana and Nigeria treatments were given at home unsupervised. In Uganda the first dose was administered as DOT.

***Information for AL was only available in the third survey conducted in 2008, so results presented are only from that survey.

#### Other combinations studied

The remaining formulations of ACT, including AS + SP, AS + MQ, dihydroartemisinin-piperaquine (DHA-PQ) and dispersible AL, have been investigated infrequently. Two studies looked at the combination AS + SP (Table 4); one study found adherence to be only 34% [30], while the other study found adherence to be twice as high at 75% [57]. In Asia, five studies reported on adherence to co-packaged AS + MQ (Table 5), with adherence reported to be >90% in four of them [26,27,50,52], while the fifth study conducted in Cambodia found adherence levels to be only 77% [27]. Two additional studies looked at adherence to ACT in general (Table 6) and found adherence rates to be less than 50% [48,53]. There were no studies found that presented adherence data on the recently released co-formulated version of ASMQ or DHA-PQ.

**Table 4:** **Studies that measure adherence to AS** + **SP**

**Study design**	**Study author**	**Country**	**Study year**	**Population**	**Sample size**	**Measurement method**	**Adherence**
**Prospective observational**	Depoortere [30]	Zambia	2002	6-59 mo	142	Pill counts & self-report (questionnaire)	39.4%
**RCT**	Kachur [62]	Tanzania	2003	<5	128	Pill counts & self-report (questionnaire) composite	75.0%

**Table 5:** **Studies that measure adherence to AS** + **MQ**

**Study design**	**Study author**	**Country**	**Study year**	**Population**	**Sample size**	**Measurement method**	**Adherence**
**Cross-sectional**	Yeung [54]	Cambodia	2002	All ages	44	Self-report	77.0%
**Prospective observational**	Congpuong [50]	Thailand	2008/2009	All ages	240	Self-report & bioassay	96.3%
	Meankaew [52]	Thailand	2009	All ages	534 total; 285*Pf*	Self-report	94.0%
	Na-Bangchang [26]	Thailand	1994/1995	All ages	126	Bioassay	98.1%*
	Shwe [27]	Myanmar	1996	All ages	380	Bioassay	99.5%**

*Full adherence reported; the majority of patients were adults.

**For both groups.

**Table 6:** Studies that measure adherence to unspecified ACT combinations

**Study design**	**Study author**	**Country**	**Study year**	**Population**	**Sample size**	**Measurement method**	**Adherence**
**Cross-sectional**	Onyango [48]	Kenya	2012*	<13	297	Self-report	47.0%
	Watsierah [53]	Kenya	2011*	<13	397	Self-report	29.4% dose 33.0% duration

*Publication year used as year of study unknown.

#### Comparative studies/multiple combinations

Seven studies compared AL to at least one other ACT or anti-malarial combination (Table 3) [39-41,55,59,60,67]. In Uganda, mean adherence to AL was 10% higher than the mean found for quinine (95% vs. 85%; p = 0.0008) [55]. In the Gambia, adherence to AL was lower than that of chlorproguanil-dapsone (CPD) (67% versus 94%; p = <0.001), however no association was found between adherence and treatment outcome for either drug [59]. In Malawi, adherence to AL was similarly compared to SP; self-reported adherence to AL or SP were both ≥ 99% and adherence as measured by MEMS was 92% vs. 90% [67]. Three studies compared AL to AQ + AS [39,40,60]. Faucher *et al*. directly compared the two combinations in Benin, and found adherence levels for co-formulated AQAS were higher (91%) than those of AL (83%), but the difference was not found to be significant (p = 0.16) [60].

### Reasons for non-adherence

Although reasons for non-adherence were not reported for all studies, there were similar trends found across the 8 studies that did report reasons for non-adherence. Four studies reported that one reason for non-adherence was that the mother/caregiver forgot to give the medication [43,58,59,63]. Three studies found that the caregiver did not understand the instructions [58,59] or gave the wrong dose of medication by giving two doses at once [44]. The limited availability of food/drink or a fatty meal/food was cited for both AL and AQ + AS as reasons for non-adherence [58,61,63]. In two studies, caregivers reported that their child was still sick after the first dose or did not improve, so the medication (AL or AQ + AS) was discontinued [43,61]. In contrast, two other studies found that the reason for non-adherence was due to the fact that the patient improved and medication was discontinued [44,63].

Furthermore, sharing or saving medications was found to be a reason for non-adherence in Ethiopia and Kenya [43,44]. In Ethiopia, Lemma *et al*. reported that patients were non-adherent to AL due to characteristics of the medication such as too many tablets, tablets were too big or bitter or that children refused to take the medication [43]. In Kenya, Ogolloa *et al*. cited that children did not like AL and were thus non-adherent [44] and Lawford *et al*. reported dislike for the medication as the reason for non-adherence [37]. Two other studies found similar findings and cited vomiting as the reason for non-adherence in children who took AQ + AS in Sierra Leone [61] and those who took AL in Uganda [63].

### Factors associated with adherence to ACT

Demographic factors, such as sex, socio-economic status or age were not significantly or consistently associated with adherence [30,31,37,38,43,45-48,54,56],[57,61-63]. However, two studies did report a significant association between age of the patient and the level of adherence [37,38]. Lawford *et al*. found both the age of the respondent (caregiver) and the age of the patient were significant factors associated with adherence in Kenya. Older caregivers (between 25–50 years of age) had 1.65 (95% CI = 1.10-1.85) the odds of being fully adherent, compared to younger caregivers (<25 years) [37]. The study also found that older patients (15+ years) were more likely to be adherent compared to those <15 years (OR = 1.37, 95% CI = 1.02-1.85). Mace *et al*. also found that younger patients (<5 years of age) in Malawi were less likely to be adherent to AL (OR = 0.05; 95% CI 0.3-0.8; p = 0.05) compared to older patients (18+ years) [38].

Education levels and literacy were both found to be significantly associated with ACT adherence in five studies, with higher levels of education and/or literacy positively associated with adherence [30,31,48,56,57]. In Zanzibar, Beer *et al*. reported that caretaker education (7+ years) was a significant predictor of adherence (OR = 5.08; p = 0.008) [56]. In Zambia, patients whose caretakers had some education had a significantly lower risk of non-adherence (RR = 0.46, 95% CI = 0.22-0.95) [30]. Similarly, in Uganda patients and/or caregivers that had attended at least some secondary school were 22% more likely to be adherent (p = 0.024) in one study [57] and in Uganda a lack of caregiver formal education had a significant association with non-adherence (OR 3.1, p = <0.05) [31]. Another study in Kenya found that higher education level (OR = 0.074, p = <0.01) and the ability to read (OR = 0.285, p = < 0.01) were both positively associated with adherence to ACT [48].

Language was also found to impact adherence. Caregivers in Uganda that could read English were found to have 0.47 fewer doses left compared to those that could not read English (p = 0.024) [57]. Depoortere *et al*. found that giving instructions on administration of treatment to caregivers in their mother tongue lowered the risk of non-adherence (RR = 0.46, 95% CI = 0.28 to 0.77). In addition, patients given the first dose as directly observed treatment (DOT) at the health centre were 2.4 times more likely to be adherent (p = 0.009) [30].

Patient/caregiver knowledge or understanding of treatment dose was found to be a significant predictor of adherence in two studies [37,63]. Patient preference or dislike for a specific drug or ACT was found to be associated with adherence in Kenya and Malawi [37,38]. And Kalyango *et al*. found that some signs and symptoms of patients such as no reported fever (OR = 3.3), caregivers’ perception that disease was not severe (OR = 2.0) and vomiting (OR = 2.6) were all found to be associate with non-adherence [63]. Achan also found that vomiting was predictor of non-adherence (p = 0.02) [55].

Mace et al. found that caregivers receiving instructions for treatment administration with a visual aide or medication package were slightly more likely to adhere to AL in Malawi (OR = 2.5, p = 0.02) [38]. Other aspects that have to do with taking or administering ACT, which have been thought to improve adherence, such as packaging doses together, providing pictorial instructions, simplicity of dosage instructions and number of pills were not prominent factors investigated. One study did report that giving the exact number of tablets for the prescribed dose was associated with adherence [56], suggesting that pre-packaged doses should improve adherence. Almost all of the patients in a Tanzanian study reported that the pictogram printed on the packages and the blister packaging depicting the correct treatment doses were helpful, but the impact of this on adherence was not assessed [36].

## Discussion

Over the past decade substantial efforts have been made to increase access and targeting of ACT for the effective management of malaria. In order to ensure that efficacious drugs are also effective in routine heath care systems, patient/caregiver adherence is important. This review summarizes the current evidence base on ACT adherence levels, adherence definitions and measurement as well as factors associated with adherence to ACT.

### Adherence levels

ACT adherence levels varied, from less than <30% for ACT in general in Kenya [53] and up to 100% adherence to AL in Malawi [67]. The lack of homogeneity in findings and the large range in adherence levels can be attributed not only to the variability between study settings, study designs and ACT formulations, but also as a result of differences in study implementation such as questionnaire/interviewing methods, blinding patients/caregivers to follow-up and study design features (e.g. RCT *vs*. observational).

For example, the questionnaire used by Kabanywanyi *et al*. to assess AL adherence in Kenya was semi-structured with open-ended questions embedded within the questionnaire [36]. Whereas the questionnaire used by Lawford *et al*. (also looking at AL adherence in Kenya) was more structured and resembled a malaria indicator survey and thus collected a different type of data [37]. Despite similar contexts and drug regimens, the findings were different with one study finding adherence to be only 64.1%, while the other found adherence to be as high as 89.2%.

Ideally, standardized, comprehensive definitions and measuring tools would be used to assess adherence, including a definition which incorporates duration, timing and frequency of dose. However, we found that this comprehensive definition was only utilized for observational studies that looked at AL, a regimen requiring multiple doses per day. In contrast, randomized controlled trials (RCTs) looked primarily at whether the drug was taken and not necessarily as to when or how it was taken.

For certain ACT formulations, such as AL, timing is important to the overall effectiveness of the regimen and should, therefore, be taken into account when determining adherence levels. From a public health perspective, a more synchronized evidence base on how and when patients take ACT can lead to more patient friendly packaging and dosing instructions. Therefore, a standardized definition of adherence would be useful to enable comparison between ACT regimens as well as to help identify contextual trends.

### Factors associated with adherence

Little is known with regard to the determinants of adherence to ACT. Findings and trends were not consistent across studies. Demographic factors, such as sex, socio-economic status or age do not seem to be factors strongly or consistently associated with adherence [30,31,37,38,43,45-48,54,56],[57,61-63]. However, it is important to note that some studies were not actually representative and/or powered to look at age groups. Two studies did find a significant association between age of the patient and the level of adherence, both suggesting that younger patients were less likely to be adherent. In Malawi, children less than five were less adherent than older children [38]; while in Kenya patients less than 15 years of age were less adherent than older patients [37].

Previously, age has been reported as a risk factor for poor adherence to non-ACT regimens [16], suggesting that age related factors should be considered when developing anti-malarial regimens and communication campaigns. Vomiting has also been found to be negatively associated with adherence to both AQ + AS and AL [61,63], however it was also considered as exclusion criteria for some studies or not accounted for when defining adherence in others. As vomiting can be influenced by severity of disease as well as treatment regimen and patient/caregiver behaviour after vomiting is influenced by knowledge provided by health workers, care should be taken when attributing non-adherence to vomiting. Further investigations surrounding vomiting and related factors and the impact on adherence is warranted.

### Study designs

As adherence is difficult to measure accurately retrospectively, the majority of studies (17) were found to be prospective observational studies. Although many of these were similar in design, differences in context and study regimes made direct comparisons challenging and precluded data synthesis.

Cross-sectional household surveys [40,70] and effectiveness studies [45,55,59,60,67], reported higher levels of adherence, however this can be attributed to the study design. In the cross-sectional surveys, adherence questions are asked retrospectively; patients or caregivers were asked to recall how they took or gave the ACT. Cross-sectional surveys are vulnerable to recall bias, particularly as the time frame for recall is often two or more weeks after receiving treatment, and thus may over- or underestimate adherence levels.

Furthermore, cross-sectional household surveys, which are often influenced by the Roll Back Malaria (RBM), Malaria Indicator Survey (MIS), focus primarily on the treatment seeking process and not on how one particular regimen was taken (dose & timing), thus offering an indication of adherence, but not an exact measurement. Additionally, patient knowledge or recognition of the drugs may not be sufficient through these types of surveys. Likewise, differences in nomenclature may play a large role in understanding the survey questions, whereby the study researchers may use the actual drug names; respondents may use local names to describe the same medication. To address this, one study carried out in Kenya made treatment charts with examples of drugs to assist respondents with their recall [48], however this was not the norm.

Prospective observational studies that interviewed patients or caregivers the day following the last treatment, should have better recall, however, the accuracy of the measurement is dependent on how patients/caregivers were recruited and whether they knew they would be followed up at a later date. In studies like that of Cohen *et al*. and Gerstl *et al*. where patients were blinded to potential follow-up, adherence levels were lower than other studies [57,61]. Souares *et al*. found similar results in Senegal, where patients were also blinded to follow-up visits after receiving treatment for amodiaquine plus sulphadoxine-pyrimethamine (AQ + SP), and reported an adherence rate of 64.7% [16]. Therefore, one could consider that participants that were aware of future follow-up visits at the time of recruitment may adhere better than those that do not.

Effectiveness study designs (RCT and pre-post designs) have similar challenges, as patients are enrolled and consent to participation prior to taking part in the study. In the majority of studies, patients/caregivers knew that they were enrolled in a study and therefore may have altered their behaviour to be more favourable (i.e. Hawthorn effect).

### Methods of measuring ACT adherence

Although studies on adherence to anti-malarials have been conducted for over a decade, methodologies and definitions of adherence still lack standardization. In a number of studies the definition for adherence was categorized as; probably adherent, probably non-adherent and non-adherent. This approach to defining adherence is crude and imprecise and may lead to an individual’s adherence status being misclassified resulting in an over- or underestimation of adherence.

Most of the methods used to measure adherence to anti-malarials were developed measuring non-ACT formulations [10], yet they are still widely used to measure adherence to ACT today. However, many of the current measurement methods used are suboptimal as malaria is typically found in countries with limited resources where patients often live in remote or hard to reach areas, which makes follow-up difficult and biological assays impractical.

Currently questionnaires for ACT adherence are not standardized and follow more complex household survey structures similar to the RBM malaria indicator survey and demographic and health surveys. For both HIV and TB treatment regimens standardized questionnaires have been used to assess treatment adherence. Some questionnaires are long and detailed (e.g. AIDS Clinical Trials Group adherence questionnaire), while others are short or abbreviated versions, which can be used during patient consultations and still provide a relatively accurate adherence measurement (e.g. the Brief Medical Questionnaire (BMQ) and Morisky Scale) [71-73]. Although questionnaires utilized for chronic disease medication adherence are not directly translatable to acute illnesses such as malaria, the idea of a short and standardized questionnaire that can be easily implemented in low resource settings would make it easier to routinely assess adherence to ACT.

Although measuring adherence through self-report is operationally less expensive and easier to implement, it is subject to social desirability bias, which may overestimate adherence. Study designs should take this into account by blinding patients/caregivers to potential follow-up visits as well as asking about medication intake in different ways during the interviews. MEMS and biological assays are more objective methods to measure adherence and may offer more precise adherence measurements, however they can both be costly and biological assays may not be possible for all ACT combinations. Furthermore, MEMS strategies may alter packaging, which may impact the way in which patients consume medications [9,13,14].

Further consensus is needed with regard to translating bioassay data into a measurement of adherence/non-adherence. Studies in this review found that biological assays were primarily incorporated into effectiveness studies looking at supervised versus unsupervised administration AL treatment with the purpose of validating whether the patient had ingested the medication. Although this method is itself objective in terms of measuring drug metabolites in the blood, interpretation of the results can be problematic. Absorption levels for lumefantrine are known to be variable due to suboptimal absorption with low fat intake [68,74,75]. None of the studies collected information on fat intake at the time of assessing adherence, thus this method may underestimate adherence levels.

Only three studies compared day-7 lumefantrine blood levels between adherent and non-adherent patients, but no study found a significant difference between the two groups [31,45,46]. Furthermore, all three studies had limited numbers of patients that were non-adherent, thus limiting their power to detect differences. For bioassays to be a viable method for measuring adherence to AL, additional studies with larger samples may be needed in order to determine if there is a correlation between blood lumefantrine levels and adherence status.

### Limitations of the review

This review has several limitations. First, information on adherence to ACT is often a secondary outcome embedded into larger studies, and details on the measurement of adherence outcomes are often missing or not reported. As a result, studies with limited information on adherence may have been missed or excluded. Second, this review was limited to peer-reviewed publications. As adherence can be considered an operational issue, much of the data collected on ACT in developing countries may be unpublished. Third, the majority of studies regarding ACT adherence (~60%) have been conducted in East or Southern Africa and the range of ACT formulations studied was narrow, with over half of the studies looking primarily at adherence to AL. Only seven studies compared adherences levels between ACT formulations, thus lacking critical information that may improve access and targeting ACT and inform policy decision- making. Fourth, as the studies were conducted in a variety of countries with different ACT combinations, amongst different age groups and populations, in different settings with different methods and sample sizes, direct comparisons of ACT adherence levels should be reviewed with caution. However, trends in adherence levels and associated factors can be noted and further explored. Finally, as adherence may be influenced by cultural and contextual factors, this review provides only a narrow picture of how ACT is taken and further qualitative investigations should be considered.

## Conclusions

This review highlights the weak evidence base on ACT adherence. Results suggest that ACT adherence levels varied substantially between study populations and comparison between studies was challenging due to differences in study design, definitions, and methods used to measure adherence. Standardising methodologies for both self-report and bioassays used for evaluating adherence of different formulations across diverse contexts would improve the evidence base on ACT adherence and effectiveness; namely, specific and measurable definitions for adherence are needed for both methodologies. Additionally, further studies of the individual factors and barriers associated with non-adherence to ACT are needed in order to make informed policy choices and to improve the delivery of effective malaria treatment.

## Abbreviations

ACT: Artemisinin-based combination therapy; AL: Artemether-lumefantrine; AMFm: Affordable Medicines Facility – malaria; AQAS: Amodiaquine-artesunate co-formulated; AQ + AS: Amodiaquine plus artesunate co-packaged; ASMQ: Artesunate-mefloquine co-formulated; AS + AS: Artesunate plus mefloquine co-packaged; AS + SP: Artesunate plus sulfadoxine-pymetheramine; BMQ: Brief medical questionnaire; CASP: Critical Appraisal Skills Programme; CONSORT: Consolidated Standards for Reporting Trials; CPD: Chlorproguanil-dapsone; DHA-PQ: Dihydroartemisinin-piperaquine; DOT: Directly observed therapy; GFATM: The global fund to fight AIDS, tuberculosis and malaria; MEMS: Medical event monitory services; MIS: Malaria indicator survey; NDOT: Non-directly observed treatment; PMI: The President’s Malaria Initiative; OR: Odds ratio; RBM: Roll Back Malaria; RR: Relative risk; RCT: Randomized Controlled Trial; STROBE: The strengthening the reporting of observational studies in epidemiology; WHO: World Health Organization.

## Competing interests

The author’s declare that they have no competing interests.

## Authors’ contributions

KB and DC conceived and designed the review. KB and ML conducted the review and synthesized the findings. KB conducted the analysis and wrote the first draft of the manuscript. KB, DC, ML and SS revised and edited the manuscript. All authors read and approved the final version of the manuscript.

## Supplementary Material

Additional file 1 **Literature review search strategy.** Table with details about the databases, key terms, limits, as well as the inclusion/exclusion criteria used for this review. Format: pdf Size: 31 KB. Click here for file

Additional file 2 **Quality assessment of studies.** This file contains three tables which showing how the quality studies were assessed. Table 1 shows the quality assessment criteria and results for Pre-/Post-Intervention and RCTs. Overall study quality was assessed to be good; however there were very few studies that incorporated blinding into the studies. Table 2 shows quality assessment criteria and results for prospective observational studies. Although the outcome definition and measurement were well defined, there were weaknesses in reporting participant selection, limited range of co-factors assessed and details about the statistical analysis, with few providing power calculations, confidence intervals or p-values. Table 4 shows quality assessment criteria and results for household cross-sectional surveys. Overall quality was assessed to be good; however information participant selection was limited particularly with regards to generalizability. Additionally, statistical details such as power calculations and refusal rates were not always reported. Format: pdf Size: 257 KB. Click here for file
