# Supplementary material for: Adherence to artemisinin-based combination therapy for the treatment of malaria: a systematic review of the evidence
Source: Malar J. 2014 Jan 6;13:7. doi: 10.1186/1475-2875-13-7 (PMC3893456; doi:10.1186/1475-2875-13-7)
Supplement: Additional file 1 — Literature review search strategy. Table with details about the databases, key terms, limits, as well as the inclusion/exclusion criteria used for this review. Format: pdf Size: 31 KB. [file 1475-2875-13-7-S1.pdf]

Additional File 1 Literature Review Search Strategy

**Databases**	1. Medline<br>2. Embase<br>3. Global Health
**Key/Search Terms**	malaria or antimalarial* or anti-malarial*<br><br>combination adj1(therap* or treat* or drug* or medic*)<br><br>Sulfadoxine-pyrimethamine or Sulfadoxine/pyrimethamine or SP or fansidar or Sulfadoxine-pyrimethamine plus Chloroquine or Sulfadoxine-pyrimethamine/Chloroquine or CQ+SP or Sulfadoxine-pyrimethamine plus amodiaquine or Sulfadoxine-pyrimethamine/amodiaquine or AQ+SP<br><br>Sulfadoxine-pyrimethamine plus mefloquine or Sulfadoxine-pyrimethamine/mefloquine or SP+MQ or Fansimef<br><br>Quinine and tetracycline or quinine/doxycycline or quinine/clindamycin<br><br>Azithromycin-Chloroquine or azithromycin+Chloroquine or Azithromycin and Chloroquine or AZ+CQ<br><br>artemisinin or artemisinin-based adj3(therap* or treat* or medic* or drug*)<br><br>amodiaquine-artesunate or amodiaquine+Artesunate or ASAQ or AQAS or Winthrop or coarsucam<br><br>Artesunate-mefloquine or artesunate/mefloquine or ASMQ or Artequin<br><br>AL or artemether-lumefantrine or artemether/lumefantrine or coartem or artemether or lumefantrine<br><br>Sulfadoxine-pyrimethamine plus artesunate or Sulfadoxine-pyrimethamine/artesunate or AS+MQ or Sulfadoxine-pyrimethamine and artesunate<br><br>dihydroartemisinin-piperaquine or DHAPQ or dihydroartemisinin/piperaquine or *Duo-Cotecxin* or *Artekin*<br><br>chlorproguanil-dapsone-artesunate or CDA or CD+A or lapdap or Artesunate/SP or artesunate-pyronaridine

	azithromycin-artesunate or azithromycin + Artesunate or AZ+AS or azithromycin and Artesunate or azithromycin/artesunate<br><br>adherence or compliance or use* or effectiveness adj2(treat* or medic* or patient or therap* or drug*)
**Limits**	
**Date**	1990-
**Language**	English
**Types of People/population**	Humans of all ages
**Location**	All regions
**Type of publication**	Peer reviewed article
**Inclusion/Exclusion Criteria**	
**Types of Studies**	Not specified
**Types of intervention**	1) Treatment of malaria with ACTs
**Exclusion Criteria**	1) No severe disease<br>2) Preventative/Chemoprophylaxis<br>3) Efficacy study<br>4) Pharm kinetics or other laboratory studies<br>5) Non *falciparum* malaria<br>6) Non-ACT studies<br>7) Other diseases<br>8) Reviews/commentaries<br>9) Other malaria related interventions
**Type of outcome measures**	• Proportion that are adherent/non-adherent
**Secondary Information**	• Definitions of adherence<br>• Measurement of adherence<br>• Factors/Determinants of adherence
**Other areas of interest**	• Impact of non-adherence<br>• Reasons for non-adherence
**When search conducted**	November 2012<br>Updated April 2013
