# Supplementary material for: Adherence to artemisinin-based combination therapy for the treatment of malaria: a systematic review of the evidence
Source: Malar J. 2014 Jan 6;13:7. doi: 10.1186/1475-2875-13-7 (PMC3893456; doi:10.1186/1475-2875-13-7)
Supplement: Additional file 2 — Quality assessment of studies. This file contains three tables which showing how the quality studies were assessed. Table 1 shows the quality assessment criteria and results for Pre-/Post-Intervention and RCTs. Overall study quality was assessed to be good; however there were very few studies that incorporated blinding into the studies. Table 2 shows quality assessment criteria and results for prospective observational studies. Although the outcome definition and measurement were well defined, there were weaknesses in reporting participant selection, limited range of co-factors assessed and details about the statistical analysis, with few providing power calculations, confidence intervals or p-values. Table 4 shows quality assessment criteria and results for household cross-sectional surveys. Overall quality was assessed to be good; however information participant selection was limited particularly with regards to generalizability. Additionally, statistical details such as power calculations and refusal rates were not always reported. Format: pdf Size: 257 KB. [file 1475-2875-13-7-S2.pdf]

Additional File 2: Quality Assessment of Studies

Table 1: Quality assessment – Pre-/Post-Intervention & RCT Studies

		**Groups**			**Blinding**			**Follow-up**		**Analysis**				
	**Authors (Year)**	*Arms*	*Random*[1]	*Balance*[2]	*Subjects*	*Investigator*	*Analysis*	*Objective*[3]	*LTFU*[4]	*Power*[7]	*Stat Methods*	*Effect size*[8]	*CI*[9]	*P-Value*[10]
**Intervention Studies**	Chinbah [1]	1	N	N	N	N	N	Y	n/a	N	N	N	N	N
	Kangwana [2]	2	Y	Y	Y	N	N	Y	n/a	Y	Y	N	Y	Y
**RCT**	Achan [3]	2	Y	Y	N	N	N	Y	Y	Y	Y	Y	Y	Y
	Asante [4]	2	N	Y	N	N	N	Y	Y	Y	Y	Y	Y	Y
	Bell [5]	3	Y	Y	N	N	N	Y	Y	Y	Y	N	N	Y
	Cohen [6]	1	N	Y	Y	N	N	Y	Y	N	Y	N	Y	Y
	Dunyo [7]	2	Y	N	N	N	N	Y	Y	Y	Y	Y	Y	Y
	Faucher [8]	3	Y	Y	N	N	N	Y	Y	Y	Y	N	N	Y
	Kachur [9]	3	Y	Y	Y	N	N	Y	Y	N	Y	Y	Y	Y
	Mubi [10]	2	Y	Y	N	N	N	Y	Y	Y	Y	Y	Y	N
	Rahman [11]	2	Y	Y	N	N	N	Y	Y	Y	Y	Y	Y	Y

[1] Allocation / sampling process described and truly random ; [2] Comparison group characteristics provided and balanced; [3]Objective measures of outcome used; [4]Numbers of subject lost to follow up provided and analysed; [5]Intention to Treat analysis used; [6]Identical follow-up in each arm; [7] Power calculation provided; [8] Measure of effect provided (e.g. OR / RR); [9] Confidence Intervals provided; [10] P-Value provided

Table 2: Quality assessment – Prospective Observational Studies

**Author (Year)**	**Selection**			**Outcome**		**Co-factors**			**Analysis**			
	Represent. [1]	*Refusals* [2]	*Power* [3]	*Definition* [4]	*Measured* [5]	*Range* [6]	*Definition* [4]	*Measured* [7]	*LTF* [8]	*Effect size* [9]	*CI* [10]	*P-Value* [11]
Congpuong [12]	N	N	N	Y	Y	N	N	N	N	N	N	N
Depoortere [13]	N	N/A	N	Y	Y	N	N	Y	Y	N	Y	N
Depoortere [14]	Y	N	N	Y	Y	N	N	Y	Y	Y	Y	N
Fogg [15]	Y	N	N	Y	Y	Y	Y	Y	Y	Y	Y	Y
Gerstl [16]	Y	Y	N	Y	Y	Y	Y	Y	Y	Y	Y	Y
Kabanywanyi [17]	Y	N	N	Y	Y	Y	Y	Y	N	N	N	N
Kalyango [18]	Y	N	Y	Y	Y	Y	Y	Y	N	Y	Y	Y
Lawford [19]	N	Y	Y	Y	Y	Y	Y	Y	Y	Y	Y	Y
Lemma [20]	Y	Y	N	Y	Y	Y	Y	Y	Y	Y	Y	N
Mace [21]	Y	Y	Y	Y	Y	Y	Y	Y	Y	Y	Y	Y
Meankaew [22]	N	N	N	N	Y	N	N	Y	N	N	N	N
Na-Bangchang [23]	N	N	N	Y	Y	N	N	N	Y	N	N	N
Ngasala [24]	Y	Y	N	Y	Y	N	N	N	Y	N	N	N
Ogolla [25]	N	N	N	Y	Y	N	N	N	Y	N	N	Y
Ratsimbasoa [26]	N	N	N	Y	Y	N	N	N	Y	N	Y	Y

Shwe [27]	N	N	N	Y	Y	N	N	N	N	N	Y	Y
Zaw Win [28]	N	N	N	Y	Y	Y	Y	N	N	N	Y	Y

[1] Sample representative of wider population of interest (selection bias); [2] Response rate provided and explained; [3] Power calculation provided; [4] Outcome / co-factor(s) clearly defined; [5]Outcome collected appropriately (misclassification bias); [6] Suitable range of variables collected; [7] Clear methods explained for collection (misclassification bias); [8] Loss to follow-up reported [9] Measure of effect provided (e.g. OR / RR); [10] Confidence Intervals provided; [11] p-values provided

Table 3: Quality assessment – Household Cross-sectional Surveys

Author (Year)	Selection				Outcome		Co-factors			Analysis			
	Sampling[1]	*Refusals*[2]	*Power*[3]	*Represent.*[4]	*Definition*[5]	*Measured*[6]	*Range*[7]	*Definition*[5]	*Measured*[8]	*Adjustment*	*Effect size*[9]	*CI*[10]	*P-Value*[11]
Ajayi [29]	N	N	N	N	Y	N	N	N	N	N	N	Y	N
Ajayi [30]	N	N	N	Y	Y	Y	N	N	N	Y	N	N	Y
Alba [31]	Y	N	N	Y	N	Y	Y	Y	Y	Y	Y	N	Y
Barnes [32]	N	Y	N	N	Y	Y	N	N	N	Y	N	N	N
Beer [33]	Y	N	Y	Y	Y	Y	Y	Y	Y	Y	Y	Y	Y
Onyango [34]	y	N	N	N	N	Y	Y	Y	Y	Y	Y	Y	Y
Simba [35]	Y	N	N	N	Y	Y	Y	Y	Y	Y	Y	Y	N
Watsierah [36]	Y	N	N	Y	Y	Y	Y	Y	Y	Y	N	N	Y
Yeung [37]	Y	Y	N	N	Y	Y	N/A	N/A	N/A	Y	Y	Y	N

[1] Simple random sampling (SRS) or reasonable alternative where SRS not possible; [2] Response rate provided and explained; [3] Power calculation provided; [4] Sample representative of wider population of interest (selection bias); [5]Outcome/co-factor(s) clearly defined; [6] Outcome collected appropriately (misclassification bias); [7] Suitable range of variables collected; [8] Clear methods explained for collection (misclassification bias); [9] Measure of effect provided (e.g. OR / RR); [10] Confidence Intervals provided; [11] p-values provided

References

1. Chinbuah AM, Gyapong JO, Pagnoni F, Wellington EK, Gyapong M: **Feasibility and Acceptability of the use of artemether-lumefantrine in the Home-management of uncomplicated malaria in children 6-59 months old in Ghana.** *Tropical Medicine and International Health* 2006, **11:**1003-1016.
2. Kangwana BP, Kedenge SV, Noor AM, Alegana VA, Nyandigisi AJ, Pandit J, Fegan GW, Todd JE, Brooker S, Snow RW, Goodman CA: **The impact of retail-sector delivery of artemether-lumefantrine on malaria treatment of children under five in Kenya: a cluster randomized controlled trial.** *PLoS Medicine / Public Library of Science* 2011, **8:**e1000437.
3. Achan J, Tibenderana JK, Kyabayinze D, Wabwire Mangen F, Kamya MR, Dorsey G, D'Alessandro U, Rosenthal PJ, Talisuna AO: **Effectiveness of quinine versus artemether-lumefantrine for treating uncomplicated falciparum malaria in Ugandan children: randomised trial.** *BMJ* 2009, **339:**b2763.
4. Asante KP, Owusu R, Dosoo D, Awini E, Adjei G, Etego SA, Chandramohan D, Owusu-Agyei S: **Adherence to artesunate-amodiaquine therapy for uncomplicated malaria in rural Ghana: a randomised trial of supervised versus unsupervised drug administration.** *Journal of Tropical Medicine* 2009, **529583**.
5. Bell DJ, Wootton D, Mukaka M, Montgomery J, Kayange N, Chimpeni P, Hughes DA, Molyneux ME, Ward SA, Winstanley PA, Lalloo DG: **Measurement of adherence, drug concentrations and the effectiveness of artemether-lumefantrine, chlorproguanil-dapsone or sulphadoxine-pyrimethamine in the treatment of uncomplicated malaria in Malawi.** *Malaria Journal* 2009, **8:**204.
6. Cohen JL, Yavuz E, Morris A, Arkedis J, Sabot O: **Do patients adhere to over-the-counter artemisinin combination therapy for malaria? evidence from an intervention study in Uganda.** *Malaria Journal* 2012, **11:**83.
7. Dunyo S, Sirugo G, Sesay S, Bisseye C, Njie F, Adiamoh M, Nwakanma D, Diatta M, Janha R, Sisay Joof F, et al: **Randomized trial of safety and**

effectiveness of chlorproguanil-dapsone and lumefantrine-artemether for uncomplicated malaria in children in the Gambia. *PLoS ONE [Electronic Resource]* 2011, **6:**e17371.

8. Faucher JF, Aubouy A, Adeothy A, Cottrell G, Doritchamou J, Gourmel B, Houzé P, Kossou H, Amedome H, Massougbodji A: **Comparison of sulfadoxine-pyrimethamine, unsupervised artemether-lumefantrine, and unsupervised artesunate-amodiaquine fixed-dose formulation for uncomplicated Plasmodium falciparum malaria in Benin: a randomized effectiveness noninferiority trial.** *Journal of Infectious Diseases* 2009, **200:**57-65.
9. Kachur SP, Khatib RA, Kaizer E, Fox SS, Abdulla SM, Bloland PB: **Adherence to antimalarial combination therapy with sulfadoxine-pyrimethamine and artesunate in rural Tanzania.** *The American Journal of Tropical Medicine and Hygiene* 2004, **71:**715-722.
10. Mubi M, Janson A, Warsame M, Mårtensson A, Källander K, Petzold MG, Ngasala B, Maganga G, Gustafsson LL, Massele A: **Malaria rapid testing by community health workers is effective and safe for targeting malaria treatment: randomised cross-over trial in Tanzania.** *PLoS ONE* 2011, **6:**e19753.
11. Rahman MM, Dondorp AM, Day NPJ, Lindegardh N, Imwong M, Faiz MA, Bangali AM, Kamal ATMM, Karim J, Kaewkungwal J, Singhasivanon P: **Adherence and efficacy of supervised versus non-supervised treatment with artemether/lumefantrine for the treatment of uncomplicated Plasmodium falciparum malaria in Bangladesh: a randomised controlled trial.** *Transactions of the Royal Society of Tropical Medicine & Hygiene* 2008, **102:**861-867.
12. Congpuong K, Bualombai P, Banmairuroi V, Na-Bangchang K: **Compliance with a three-day course of artesunate-mefloquine combination and baseline anti-malarial treatment in an area of Thailand with highly multidrug resistant falciparum malaria.** *Malaria Journal* 2010, **9:**43.
13. Depoortere E, Salvador ETC, Stivanello E, Bisoffi Z, Guthmann JP: **Adherence to a combination of artemether and lumefantrine (Coartem)**

in Kajo Keji, southern Sudan. *Annals of Tropical Medicine & Parasitology* 2004, **98:**635-637.

14. Depoortere E, Guthmann J-P, Sipilanyambe N, Nkandu E, Fermon F, Balkan S, Legros D: **Adherence to the combination of sulphadoxine-pyrimethamine and artesunate in the Maheba refugee settlement, Zambia.** *Tropical Medicine & International Health* 2004, **9:**62-67.
15. Fogg C, Bajunirwe F, Piola P, Biraro S, Checchi F, Kiguli J, Namiiro P, Musabe J, Kyomugisha A, Guthmann J-P: **Adherence to a six-dose regimen of artemether-lumefantrine for treatment of uncomplicated Plasmodium falciparum malaria in Uganda.** *American Journal of Tropical Medicine & Hygiene* 2004, **71:**525-530.
16. Gerstl S, Dunkley S, Mukhtar A, Baker S, Maikere J: **Successful introduction of artesunate combination therapy is not enough to fight malaria: results from an adherence study in Sierra Leone.** *Transactions of the Royal Society of Tropical Medicine & Hygiene* 2010, **104:**328-335.
17. Kabanywanyi AM, Lengeler C, Kasim P, King'eng'ena S, Schlienger R, Mulure N, Genton B: **Adherence to and acceptability of artemether-lumefantrine as first-line anti-malarial treatment: evidence from a rural community in Tanzania.** *Malaria Journal* 2010, **9:**48.
18. Kalyango JN, Rutebemberwa E, Karamagi C, Mworozi E, Ssali S, Alfven T, Peterson S: **High Adherence to Antimalarials and Antibiotics under Integrated Community Case Management of Illness in Children Less than Five Years in Eastern Uganda.** *PLoS ONE* 2013, **8:**e60481.
19. Lawford H, Zurovac D, O'Reilly L, Hoibak S, Cowley A, Munga S, Vulule J, Juma E, Snow RW, Allan R: **Adherence to prescribed artemisinin-based combination therapy in Garissa and Bunyala districts, Kenya.** *Malaria Journal* 2011, **10:**281.
20. Lemma H, Lofgren C, San Sebastian M: **Adherence to a six-dose regimen of artemether-lumefantrine among uncomplicated Plasmodium falciparum patients in the Tigray Region, Ethiopia.** *Malaria Journal* 2011, **10:**349.
21. Mace KE, Mwandama D, Jafali J, Luka M, Filler SJ, Sande J, Ali D, Kachur SP, Mathanga DP, Skarbinski J: **Adherence to treatment with artemether-**

lumefantrine for uncomplicated malaria in rural Malawi. *Clinical Infectious Diseases* 2011, **53:**772-779.

22. Meankaew P, Kaewkungwal J, Khamsiriwatchara A, Khunthong P, Singhasivanon P, Satimai W: **Application of mobile-technology for disease and treatment monitoring of malaria in the.** *Malaria Journal* 2010, **9:**237.
23. Na-Bangchang K, Congpuong K, Sirichaisinthop J, Suprakorb K, Karbwang J: **Compliance with a 2 day course of artemether-mefloquine in an area of highly multi-drug resistant Plasmodium falciparum malaria.** *British Journal of Clinical Pharmacology* 1997, **43:**639-642.
24. Ngasala BE, Malmberg M, Carlsson AM, Ferreira PE, Petzold MG, Blessborn D, Bergqvist Y, Gil JP, Premji Z, Martensson A: **Effectiveness of artemether-lumefantrine provided by community health workers in under-five children with uncomplicated malaria in rural Tanzania: an open label prospective study.** *Malaria Journal* 2011, **10:**64.
25. Ogolla JO, Ayaya SO, Otieno CA: **Levels of Adherence to Coartem© In the Routine Treatment of Un-complicated Malaria in Children Aged Below Five Years, in Kenya.** *Iranian Journal of Public Health* 2013, **42:**129-133.
26. Ratsimbasoa A, Ravony H, Vonimpaisomihanta J-A, Raherinjafy R, Jahevitra M, Rapelanoro R, Rakotomanga JDDM, Malvy D, Millet P, Menard D: **Compliance, safety, and effectiveness of fixed-dose artesunate-amodiaquine for presumptive treatment of non-severe malaria in the context of home management of malaria in Madagascar.** *American Journal of Tropical Medicine & Hygiene* 2012, **86:**203-210.
27. Shwe T, Lwin M, Aung S: **Influence of blister packaging on the efficacy of artesunate + mefloquine over artesunate alone in community-based treatment of non-severe falciparum malaria in Myanmar.** *Bulletin of the World Health Organization* 1998, **76 Suppl 1:**35-41.
28. Zaw Win T, Zaw L, Khin W, Khin L, Myitzu Tin O, Thar Tun K, Kyaw Zin T: **Adherence to the recommended regimen of artemether-lumefantrine for treatment of uncomplicated falciparum malaria in Myanmar.** *Myanmar Health Sciences Research Journal* 2012, **24:**48-53.
29. Ajayi IO, Browne EN, Bateganya F, Yar D, Happi C, Falade CO, Gbotosho GO, Yusuf B, Boateng S, Mugittu K, et al: **Effectiveness of artemisinin-**

based combination therapy used in the context of home management of malaria: A report from three study sites in sub-Saharan Africa. *Malaria Journal* 2008, **7**.

30. Ajayi IO, Browne EN, Garshong B, Bateganya F, Yusuf B, Agyei-Baffour P, Doamekpor L, Balyeku A, Munguti K, Cousens S, Pagnoni F: **Feasibility and acceptability of artemisinin-based combination therapy for the home management of malaria in four African sites.** *Malaria Journal* 2008, **7**.
31. Alba S, Hetzel MW, Goodman C, Dillip A, Liana J, Mshinda H, Lengeler C: **Improvements in access to malaria treatment in Tanzania after switch to artemisinin combination therapy and the introduction of accredited drug dispensing outlets - a provider perspective.** *Malaria Journal* 2010, **9:**164.
32. Barnes KI, Durrheim DN, Little F, Jackson A, Mehta U, Allen E, Dlamini SS, Tsoka J, Bredenkamp B, Mthembu DJ, et al: **Effect of artemether-lumefantrine policy and improved vector control on malaria burden in KwaZulu-Natal, South Africa.** *PLoS Medicine / Public Library of Science* 2005, **2:**e330.
33. Beer N, Ali AS, Rotllant G, Abass AK, Omari RS, Al-mafazy A-wH, Bjorkman A, Kallander K: **Adherence to artesunate-amodiaquine combination therapy for uncomplicated malaria in children in Zanzibar, Tanzania.** *Tropical Medicine & International Health* 2009, **14:**766-774.
34. Onyango EO, Ayodo G, Watsierah CA, Were T, Okumu W, Anyona SB, Raballah E, Okoth JM, Gumo S, Orinda GO: **Factors associated with non-adherence to Artemisinin-based Combination Therapy (ACT) to malaria in a rural population from holoendemic region of western Kenya.** *BMC Infectious Diseases* 2012, **12:**143.
35. Simba DO, Kakoko D, Tomson G, Premji Z, Petzold M, Mahindi M, Gustafsson LL: **Adherence to artemether/lumefantrine treatment in children under real-life situations in rural Tanzania.** *Transactions of the Royal Society of Tropical Medicine & Hygiene* 2012, **106:**3-9.
36. Watsierah CA, Jura WGZO, Raballah E, Kaseje D, Abong'o B, Ouma C: **Knowledge and behaviour as determinants of anti-malarial drug use in a peri-urban population from malaria holoendemic region of western Kenya.** *Malaria Journal* 2011, **10:**99.

37. Yeung S, Van Damme W, Socheat D, White NJ, Mills A: **Access to artemisinin combination therapy for malaria in remote areas of Cambodia.** *Malaria Journal* 2008, **7:**96.
